# New York Academy of Medicine.—Section on Orthopedic Surgery

**Published:** 1891-07

**Authors:** 


### NEW YORK ACADEMY OF MEDICINE.—SECTION ON ORTHOPEDIC SURGERY.

*Stated Meeting, April 17, 1891.*

Samuel Ketch, M. D., Chairman.

### OBSCURE DISEASE OF THE HIP.

Dr. H. L. Taylor presented for diagnosis the case of a boy who was taken suddenly sick, without known cause, on September 12th last, with a chill and irregular fever, which was soon followed by severe pain and swelling about the upper part of the thigh, especially in front. He was examined late in November, when he presented hardness and swelling of the upper part of the thigh with some edema, and, at times, below the knee; there was fluctuation on the outer aspect of the thigh, five inches below the anterior superior spine, and a tablespoonful of serum was several times withdrawn. The thigh was extremely flexed, abducted and everted, and there was but little hip motion, but no muscular spasm. The patient improved in health rapidly, the inflammatory symptoms subsided, and the deformity was reduced to a moderate one, by rest in bed with gentle counter-extension. The patient now walked with a considerable limp, but without support. There was a large, hard swelling connected with the right side of the pelvis, found on pressing above Poupart's ligament, and also above and in front of the hip. The patient's health was good.

Dr. A. B. Judson thought that the abduction and fixation present, indicated articular osteitis of the hip, obscured in this case by unusual deportment of the abscesses.

The Chairman thought it was very unusual for hip disease to come on with such sub-acute symptoms, and the history seemed to point rather to some infectious disease than to a chronic joint lesion.

### KNEE- AND ANKLE-JOINT DISEASE IN INFANTS.

Dr. Royal Whitman presented a series of cases illustrating the treatment of knee- and ankle-joint disease in young infants, by an adaptation of Thomas's splint. He claimed that by the routine use of extension, not merely for its effect on the contracted knee and as a factor in the production of rest, but for the purpose of retaining the brace in position, instead of using the ordinary shoulder strap, the constant shifting of the ring, which occurs in infants, is avoided, and hence the irritation of the skin, and motion at the diseased joint, were reduced to a minimum. He advised the use of the brace in cases of ankle-joint disease, even when the infant had not begun to walk, on the principle that children long before they walk are making constant attempts to stand, and in creeping or otherwise, expose the joint to injury.

The brace is made of light material, with two leather straps attached to the foot-bar. The extension plasters are applied to the leg, usually below the knee, and the leg is firmly bandaged from the toes to the groin, and the brace applied with sufficient extension to hold the ring firmly in its place. The leg and brace are then firmly bandaged to one another, from the foot to the ring. Such an arrangement provides rest, compression, and protection, the effect of which in painful, contracted and suppurating joints is at once apparent. In ankle-joint disease, the foot being at a right angle to the leg, a well-fitting plaster bandage is first applied.

It seemed to the speaker that the treatment of joint disease in infancy should be carried out on the same principles as apply to older children, and that in cases of knee- and ankle-joint disease the conditions are most satisfactorily met by the Thomas brace. With ordinary care, its use was attended with no difficulty, and it was not unusual to see infants of from fifteen to eighteen months of age, walking about on the brace and high shoe without discomfort.

Dr. V. P. Gibney said that his clinical experience had taught him that it was almost impossible to hold the limb down with plaster-of-Paris in these small children; for, notwithstanding the performance of tenotomies, as soon as the plaster becomes soft, which it will do speedily as a result of the dribbling of the urine, and from other causes, the limb will begin to flex again. This adaptation of the Thomas splint he considered an admirable one.

Dr. John Ridlon said that this method of employing traction had been used by Thomas years ago, but more recently that surgeon had preferred to straighten the cases with or without an anesthetic, and then put them up permanently in a straight position with absolute immobilization, and with as much traction as could be obtained by a full-length caliper splint. He thought that direct backward pressure, with a pad above and below the knee, and a strap behind the knee, was better than the method of bandaging adopted in one of the cases presented. For the ankle-joint cases, he thought a metal splint was more satisfactory than the plaster-of-Paris. He had repeatedly tried plaster-of-Paris in these cases, and had found that it failed to keep the limb straight.

Dr. Judson doubted whether in larger children the splint shown could be relied on to secure both fixation and protection.

Dr. A. M. Phelps disagreed entirely with Dr. Ridlon as to the superiority of a metal splint over the plaster-of-Paris. He had used the Thomas splint for the past four years as a protection to the joint, but he did not approve of producing extension by it, as this caused intra-articular pressure. His plan was to reduce the deformity under ether, and then apply plaster-of-Paris. He did not think the splint exhibited was any better than plaster-of-Paris for small children before they began to walk.

Dr. W. R. Townsend said that it was because they had experienced so much trouble from excoriations in the use of plaster-of-Paris at the hospital, that Dr. Whitman had devised this arrangement.

Dr. N. M. Shaffer saw no necessity for either this splint or for plaster-of-Paris. There had been no trouble from the apparatus which he had employed in his practice, and he thought it gave even better protection than the Thomas splint. The deformity is usually made too important a factor in the treatment. A study of Nature's methods would show that the deformity should be reduced by modifying rather than by increasing the intra-articular pressure, and Dr. Whitman committed an error in attempting to reduce the deformity quickly; for, in the majority of cases, Nature endeavored to warn us against this rapid reduction of the deformity by establishing a condition of muscular resistance. The slow method of reducing the deformity, in his opinion, gave better ultimate results.

The Chairman endorsed the views of the previous speaker. Some of these cases had been stated not to have had reflex spasm, but he could not understand how this could be the case, as, in his experience, spasm had been invariably present. The apparatus acted upon the principle of the simple perineal crutch, and only emphasized the necessity for using this crutch in all these cases.

Dr. Whitman, in closing, said that the only claim to originality which he made, was in the manner of holding the brace firmly against the groin. He was fully convinced that plaster-of-Paris was very undesirable for young infants, as the joint was often swollen to the size of the thigh, and the plaster speedily worked down and became loose. However beautiful might be the theory of increasing the intra-articular pressure by traction made with this splint, the fact still remained, that while the limb was being brought down these children were comfortable, their general condition improved, and the joint diminished in size. The bandaging to which Dr. Ridlon alluded was not for the purpose of straightening the leg, but to hold the brace firmly. He considered that the deformity *was* of much importance, and that the sooner it was reduced, especially in knee disease, the better. If seen moderately early, one could be sure that recovery without deformity would occur, with the exception of some shortening.

### ANTERIOR POLIOMYELITIS.

Dr. Gibney presented a case of anterior poliomyelitis, which had so affected the abductor group of muscles as to cause marked deformity of the limb. When six years old, the patient was reported to have had a high fever, accompanied by inability to move the legs. This condition rapidly improved, and then it was noticed that the child could use the limbs but little. He had first seen the case on November 16, 1887, at which time there was complete adduction of the limb, the hamstrings were considerably shortened, and there was some flexion at the hip. Shortly after he began treatment by stretching the hamstrings, the case passed under the care of a distinguished general surgeon, who presented her to the Surgical Society as a case of congenital dislocation of the hip. She returned after a time, and the extension was resumed. On

November 22, 1889, she was anesthetised, and, by an open incision over the tensor vaginæ femoris and flexors of the thigh, he was able to divide freely the contracted tissues, until the limb could be brought into good position, when he applied plaster-of-Paris from the axilla to the toes. After two or three weeks, a brace was applied. On October 22d, another operation was necessary. On March 26, 1890, the limbs were parallel, and at present it is difficult to produce any luxation of the hip-joint, the limbs are of equal length, and the child can walk fairly well without any apparatus. He thought the case showed the advantages of protecting the weakened muscles for a long time.

Dr. SHAFFER said that he wished to emphasize the important part played by the tensor vaginæ femoris, this muscle and the sartorius often being the principal opponents to good locomotion. He had seen several cases in which the general surgeon had made a diagnosis of dislocation of the hip, owing to the extreme malposition of the thigh. Division of the tensor vaginæ femoris, and of the muscles attached to the anterior spine of the ilium, is the only method of treating these cases successfully, and, in order that the division be thorough, he preferred the open method to the subcutaneous.

### ARREST OF DEVELOPMENT.

Dr. GIBNEY presented two cases: In the first, in addition to some deformity of the hands, the patella and knee joints were rudimentary. There was limitation of motion and rotation inwards of the femur, and outward rotation of the tibia, and the patella was displaced to the outer side of the femur. There was the usual marked degree of knock-knee, and the double congenital equino-varus. She was admitted to the hospital on June 18, 1889; at that time she was eight years of age, and in good general condition. After a number of tenotomies and other minor operations, it was found that although there was some improvement, there was still marked knock-knee, and obstinate equinus. Accordingly, on October 28, 1890, the astragalus and a portion of the cuboid were removed from the right foot, and the same operation was performed on the left foot on January 19th of the present year. She is now walking with apparatus, but bids fair to have a good pair of limbs.

The second case was admitted on September 20, 1887, and, on January 1, 1888, chloroform was administered, when the tendo

Achillis was divided. It was subsequently treated by stretching, but the deformity recurred, and on November 7, 1890, the greater part of the astragalus was removed. She is now doing well.

Dr. PHELPS congratulated Dr. GIBNEY on the very excellent results which he had obtained, and remarked that they seemed to demonstrate the superiority of this method of treatment by the removal of the astragalus.

### CLUB-FOOT.

Dr. GIBNEY also presented a case of club-foot, remarking that he never felt sure of having thoroughly relieved the deformity until a condition of marked calcaneus had been secured before the patient was discharged from the hospital.

Dr. SHAFFER said that hyperextension *immediately* after the operation of tenotomy was not unattended by risk, for one case of severe equino-varus which he had treated in this way, resulted in an elongated tendon.

Dr. R. H. SAYRE said that he had called attention in a previous paper to the fact that it was not wise to put the foot in a position of complete extension, because it is likely to result in too long an attachment between the calf muscles and the foot. The best position was at right angles to the long axis of the tibia. Non-union was most commonly due to the bandages being applied so tightly that the space between the divided tendon is occluded. If this fault in dressing be avoided, there is always a sufficient bond of union, even though the space be three inches long, as it was in a case which he had already exhibited to the Section.

Dr. PHELPS said he agreed thoroughly with Dr. Gibney as to the advantages of immediate hyperextension. It was the subsequent use of traction machines that pulled out the tendons into thin bands. The space between the divided ends of the tendon is immaterial, so long as the operation be performed antiseptically, and the dressings carefully applied. He had practised over-correction in 161 cases of open incision, and in not a single instance did the tendo Achillis fail to unite.

### A CASE OF NEUROMIMESIS.

Dr. W. R. TOWNSEND presented such a case. A girl, fourteen years of age, having a good family history, fell on the 27th of last January, twisting her foot, and producing a slight excoriation on the ankle. She was taken to a hospital, where the injury was treated by plaster-of-Paris for five weeks. On removing the plaster, the foot was found to be much distorted. She then came under the speaker's care, and an examination by Dr. B. Sachs indicated that the deformity was entirely due to psychical causes. There is now a slight equinus, and the extreme contraction of the tibialis produces varus. Only slight force is required to bring the foot into the normal position, and the patient can so retain it by the power of the will, for a few moments. There had been but little improvement so far in the case, which had been treated only by the application of blisters to the lower end of the spine, and by the administration of tonics.

In answer to a question from Dr. Ridlon, Dr. Townsend said that the genitals had not been examined.

Dr. Ridlon said that he asked this question because, in a recent case, a vulvitis has seemed to be the cause of the trouble.

Dr. Shaffer said that some time ago he had presented a somewhat similar deformity of the foot, but in his case there was a rhythmical action of the muscles of the thigh.

Dr. H. L. Taylor thought the diagnosis was unquestionably correct. Some years ago he had had a very similar case, which began with a slight sprain, and which completely cured in about one month.

Dr. R. H. Sayre called attention to the remarkable resemblance which this purely muscular deformity bore to that seen in cases which are considered to be incurable except by the removal of considerable portions of bone. In this case, the bony prominences are marked, and yet the bones are not luxated.

Dr. H. W. Berg spoke of the possible medico-legal interest that might attach to such cases.

### INFANTILE HERNIA.

Dr. Charles N. D. Jones, of Brooklyn, presented a child upon whom he had operated for infantile hernia. When the child was about three months old, a swelling appeared in his left groin, and gradually extended downward, until it finally reached the scrotum. The mother noticed that the swelling increased along the course of the inguinal canal, when the child made any violent effort which brought into play the diaphragm or abdominal muscles. Some weeks later, the child was fitted with a truss, but the instrument did not prevent the reappearance of the swelling in the scrotum.

In this case, as persistent efforts during the past year had failed to retain the hernia in situ, it was decided to close the canal by operative measures. The operation was done on December 20, 1890. After a thorough cleansing of the field of operation, the patient was anesthetised, the bowel reduced within the abdomen, and an incision made sufficiently large to expose the external abdominal ring. After the preliminary incision, the operator had found, contrary to his expectation, that the sac was distinct from the tunica vaginalis. It was carefully separated from the testicle and cord, and then opened transversely about an inch above the distal extremity. After a careful exploration to see that there were no adhesions, the sac was ligatured with catgut, and removed. The pillars of the ring were then brought together with catgut sutures, leaving sufficient space for the passage of the cord. The wound was packed with iodoform gauze, and treated by the open method. Convalescence was afebrile. The child, who was three years of age, was presented to the Section. The wound was firmly closed, and there was no tendency to a recurrence of the hernia. A tight phimosis was operated upon at the same time as the hernia.

### DOUBLE SUPRA-CONDYLOID OSTEOTOMY OF THE FEMURS.

Dr. Jones also presented a boy, six years of age, who was admitted to the Children's Hospital on September 10, 1890. He had a rachitic history, and all the bones presented rachitic deformity The teeth were deficient, and the femora presented anterior and lateral curvatures, with great depression of the internal condyles. Below the knee, in both legs, there was a marked anterior and inward angular deformity of both bones of the leg.

On October 21, 1890, he performed supra-condyloid osteotomy of both femora. The wounds were dressed antiseptically, put up in plaster splints, and suspended by weights and pulleys, as recommended in an article published in the *Annals of Surgery*, April, 1889. On November 15th, he performed cuneiform osteotomy on the tibia and fibula of both legs for the correction or the principal deformity. The wounds were dressed according to the method recommended by Von Bergmann—viz., they were thoroughly packed with iodoform gauze, dressed antiseptically, and left until the following day, when, all hemorrhage having ceased, the bones were united with catgut sutures, the periosteum and the skin wounds sutured, and the limb enveloped in a mass of sublimate gauze. Plaster bandages and suspension were then applied as before. Recovery was uninterrupted. On January 9th, an additional section of the bones of both legs was made to correct a slight remaining deformity, and the same after-treatment was adopted. The patient presented a very tight and adherent prepuce, which was a constant source of irritation to him. At the first operation he was circumcised. This apparently slight operation the speaker considered important, as he had found it necessary to perform it in the case of every deformity in a male child which had come under his observation.

THE PLACE OF FIXATION IN THE TRACTION TREATMENT OF HIP DISEASE.

This was the title of a paper by Dr. Robert W. Lovett, of Boston, who illustrated his remarks by the exhibition of apparatus. He said that it was a question for those who believe in the traction treatment of hip disease, to consider whether apparatus should have as its object simply traction, or fixation of the joint as well. This question presents itself under two aspects :

(*a*) As to the advisability of using, in certain cases, a splint which should give better fixation than the long traction splint; and (*b*) the indications for fixation in bed, and the class of cases in which it is necessary.

The long traction splint was introduced under the impression that it was an appliance which should give motion without friction. Later, traction in itself came to be regarded as a means of fixation, and Dr. Judson was the upholder of the view that traction furnished fixation to the hip-joint. Some experiments by the writer tended to prove that the long traction splint was not a fixation appliance, and one worn by a boy with normal hip-joints, allowed motion of 35° in walking and sitting. The practical question arises, whether such a splint furnished enough fixation, or whether in certain cases more perfect fixation of the joint is not to be desired. Certain cases do badly under treatment by the long traction splint, and these seem to be of two sorts,—very severe cases; and cases where the patients are under imperfect control, and run and play continually, producing traumatism of the joint, which results in sensitiveness, irritability, and malposition.

In the hope of preventing this condition in such cases, a splint was shown which was practically a combination of the Taylor and Thomas splint. The appliance fixes the thorax, the pelvis, and the leg, and comes below the foot, ending in a traction appliance, in this way fixing the hip-joints as perfectly as possible, and at the same time making traction upon the diseased limb. The writer would advocate the use of such a splint chiefly in hospital practice, in very severe cases, and in patients under imperfect parental control. Practical experience has shown the splint to be useful in this class of cases.

(*b*) With regard to the second division of the subject—fixation in bed—the experience of the Boston Children's Hospital has been, that the immediate treatment of malposition or joint sensitiveness results in a very small proportion of abscesses among the cases treated. Of 183 cases admitted in the last three years, 107 were sent to the wards on account of deformity and sensitiveness, and only 52 for abscess. In these years, the percentage of cases admitted for deformity and sensitiveness has steadily increased, while the percentage admitted for abscesses has steadily diminished. In the last six years at the Children's Hospital, among 574 new cases of hip diseases coming in that time, only 107 abscesses have developed, giving a percentage of 18.7 per cent., which is very much less than any other series of cases reported. Of Dr. Gibney's cases, sixty per cent. had abscess; in the Clinical Society's cases, sixty-nine per cent.; and in the recent cases of Mr. Marsh, fifty per cent. It has seemed that the early admission of cases was to be regarded as the preventive treatment of abscess. It would seem, therefore, that the use of a splint affording more fixation than the ordinary traction splint was needed in severe and sensitive cases, and that rest in bed was advisable when malposition occurs, not only in order to overcome the malposition, but in the hope of preventing abscess.

DISCUSSION.

Dr. Ridlon approved of the author's observations upon the traction splint, but the outline of the splint which he had exhibited was certainly improper. During the last few years, he had not found occasion to employ more traction than was obtained by the tendency of the Thomas splint to work downward. If the splint were not supported by shoulder-straps, it gave sufficient traction for the successful treatment of fracture of the upper part of the thigh bone. He questioned very much the advisability of allowing the patient to walk around, who had sufficient muscular spasm to indicate the necessity for the application of a special traction apparatus.

Dr. Shaffer thought that the author's experiments to determine the amount of motion occurring at the joint were fallacious, as they did not take into account the considerable arc of motion produced by the flexibility of the lumbar spine. He thought that his own experiments upon this point had not yet been contradicted. In these, he applied the apparatus to a healthy hip-joint on a person whose opposite joint was anchylosed. A person with an anchylosed hip can walk, or even dance, owing to the flexibility of the neighboring parts.

Dr. Judson thought that the traction splint secured fixation, but not immobilization. He thought it was important to make this distinction. Immobilization is found in union after fracture, and in anchylosis, while fixation is produced by reflex muscular action and by traction. It is almost impossible to immobilize a joint by any application of mechanical surgery. Fixation implies a degree of mobility which allows a reduction of the deformity. When applied in a painful case, it has a wonderful effect in relieving the patient's distress, which is partly pain and partly a sleep-destroying apprehension of disturbance of the joint.

Dr. Phelps said that as he believed that it was a cardinal principle in the treatment of all joint disease, that the affected part should be immobilized, he could not understand what the author meant by "motion within certain limits"; he saw no reason for the joint being moved at all. During the period of pain, we all agreed that rest in bed was the proper thing, and yet, if this represented the best method of treatment, why employ a splint which would not carry out this idea? More than 75 per cent. recovered without deformity. Again, if extension were the proper thing, why not counteract the action of the abductors and adductors which cause the spasm, by making use of lateral traction? He did not think the statistics about abscess collected by the author carried much weight, because, in Boston, these cases were sent to institutions at an earlier stage of the disease than they were here.

The Chairman referred to an article by Dr. Judson, in which it was shown quite conclusively that the effect of mechanical treatment, when applied sufficiently early, was to prevent abscess, and that it even prevented the opening of many abscesses which had already formed at the time the treatment was begun. Long before the Thomas splint, or the lateral traction splint, were known here, Drs. Sayre and Davis obtained cures without deformity, by means of the traction apparatus commonly employed, and he would not, therefore, accept the view that almost all the cases treated by this much-abused traction splint, pursued an unfavorable course, and ended in deformity.

Dr. Berg spoke in favor of the use of apparatus which did not require any elaborate fitting; for, as he said, "some braces require so much fitting that they rarely fit."

Dr. H. L. Taylor said that he was glad to be able to approve nearly all the points made by the author in his excellent paper. The hip-joint required some form of fixation as well as extension when acutely inflamed. In most cases, the amount of fixation afforded by the long counter-extension splint, combined with short periods of rest in bed, when necessary, was sufficient. Dr. C. Fayette Taylor had never claimed that his long splint gave positive immobilization of the hip, but the speaker was surprised at the range of motion found under its use by Dr. Lovett, and would wait for further experiment before admitting that the question of the amount of motion allowed was settled. In very bad or unruly cases in dispensary practice, the apparatus shown by Dr. Lovett would, no doubt, prove useful. The speaker would emphasize the advantage of properly-applied counter-extension in the progressive stage of hip-joint disease, in order to restore the hygiene of the joint and prevent deformity.

Dr. Lovett said that he had used one perineal pad, instead of two, because his object had been to find the fixative power of traction, and not that of any special splint, and he thought that his experiments, as far as they had gone, were in the proper direction. With regard to the question of abscess, he should have added that 170 cases of abscess mentioned included those occurring in cases which had been admitted for a number of years past,—at least since 1880.

### UPPER DORSAL KYPHOSIS.

Dr. T. Halsted Myers presented a specimen showing an upper dorsal kyphosis with the cord in situ. The patient had muscular weakness of the legs, and exaggerated knee-jerks only. The specimen showed that the pressure was made by the body of one vertebra, and that if a laminectomy were done, the arches of at least four vertebræ would have to be removed. It also showed that the pressure was entirely anterior, and that, therefore, as there was considerable room posteriorly, the operation would not benefit this patient. He thought a sharp bend in the cord, even without direct external pressure, might cause vascular changes from the increased pressure on the concave side, sufficient to cause symptoms.

Dr. SAMUEL LLOYD said that in many of the cases which had been operated upon, sufficient bone had not been removed, and that this had been the difficulty with two of Kraska's cases. As a matter of fact, it had been found that the removal of the posterior portion of the spinal column—the laminæ—*did* relieve the pressure on the cord.

